# Is There any Concordance between of IHC with FISH in HER2-Positive Breast Cancer Patients?

**Published:** 2017-01-01

**Authors:** Mehrdad Payandeh, Masoud Sadeghi, Edris Sadeghi, Alireza Janbakhsh

**Affiliations:** 1 Department of Hematology and Medical Oncology, Kermanshah University of Medical Sciences, Kermanshah, Iran; 2 Students Research Committee, Kermanshah University of Medical Sciences, Kermanshah, Iran; 3 Medical Biology Research Center, Kermanshah University of Medical Sciences, Kermanshah, Iran; 4 Department of Infectious Diseases and Tropical Medicine, Kermanshah University of Medical Sciences, Kermanshah, Iran

**Keywords:** FISH, Hormone receptors, DFS, Trastuzumab

## Abstract

**Background** **: **In developed or developing countries, the most common cancer in women is breast cancer with a pick in 40–50 years in Asia. Herein, we compared the association between IHC with FISH in HER2-positive breast cancer patients and affection of trastuzumab on disease free survival and overall survival (OS).

**Subjects and Methods:** Immunohistochemical (IHC) analysis of hormone receptors and HER2 was performed in 133 patients with breast cancer between 2003 and 2014. Patients were selected for Herceptin adjuvant treatment, according to IHC 3+ or FISH+. The specimens for pathology reports were fixed at 10% neutral-buffered formalin (pH=7.4) for 24 hours, then sliced into 4 μm sections.

**Results:** The mean age of patients at diagnosis was 46.39 years (range, 24-78 years), 100% female. Concordance rates between IHC and FISH were 31.1% for IHC 2+ and 84.1% for IHC 3+ (p<0.001). The 87 patients had age ≤50 years and 46 patients had >50 years. Of the 133 patients, 30 patients (22.6%) had metastasis and 72 (54.1%) had right involvement. Ninety three (69.9%) patients had lymph node invasion. 48 patients (36.1%) were treated with trastuzumab and 85 (63.9%) were treated without trsastuzumab. The 10-year survival rate was 70% and the mean survival was 49 months.

**Conclusion:** We recommend clinicians that FISH analysis is as a predictor in breast cancer patients with IHC score 2+. In contrast, FISH analysis of IHC 3+ samples was no useful. Trastuzumab therapy is effective and tolerated for breast cancer with IHC 3+ and probably IHC 2+/FISH+.

## Introduction

 Breast cancer is the most common cancer (27% of all cancers) and common cause of death (16%) in around the world.^1^ In Asia, maximum incidence rate is in 40–50 age groups and in contrast, in western countries the increases incidence increases with age.^2^ The human epidermal growth factor receptor 2 (HER-2/neu) or (cerbB-2) gene is located on human chromosome 17q21 and is a member of ErbB family of receptor tyrosine kinases.^3^^,^^4^ Over-expression of HER-2/neu gene and its protein are associated with disease prognosis and treatment.^3^

Trastuzumab (herceptin®), a recombinant, humanized, monoclonal antibody targeting HER2 is well established as an effective treatment for HER2-positive breast cancer.^5^^,^^6^ Hormone receptors, including the estrogen receptor (ER) and progesterone receptor (PR) status are key molecules in breast cancer.^7^^,^^8^ Protein over-expression detected by immunohistochemical (IHC) or amplification of Her-2 gene analyzed by fluorescence in situ hybridization (FISH) are two main methods used to detect Her-2 status in clinical practice.^8^ IHC is a preferred method for screening and determining cases which need to be genetically evaluated.^9^ Herein, we compared the association between IHC with FISH in HER2-positive breast cancer patients, and affection of trastuzumab on disease free survival (DFS) and overall survival (OS).

## SUBJECTS AND METHODS

 This study was approved by Ethics Committee of Kermanshah University of Medical Sciences, Kermanshah, Iran (Ethical code number: KUMS.REC.1394.493). Between 2003 and 2014, 133 breast cancer patients with HER2-neu positive in IHC test were referred to our clinic. The patients were categorized on the basis of IHC values as +2 or +3. Only patients with IHC 3+ and FISH+ were selected for herceptin adjuvant treatment and were centrally reviewed ER, PR and HER2 copy numbers. The magnitude of trastuzumab benefit was assessed using the Cox proportional hazards model for the DFS and the OS. 

The specimens for pathology reports were fixed at 10% neutral-buffered formalin (pH=7.4) for 24 hours, then sliced into 4-μm sections. Her-2 protein expression was measured using a commercial available S-P kit. FISH for Her-2 gene amplification was performed in laboratory of Taleghani Hospital using a commercial available double-color probe. Monoclonal antibodies against ER, PR, HER2, as well as IHC kit were purchased from Pars Azmon Co. Equivalent phosphate-buffered saline (PBS) was used as a negative control for primary antibodies.

**Statistical analysis**

The OS was calculated using Kaplan-Meier method and the DFS and the OS with log-rank test. The OS is defined as time from randomization until death from any cause or endpoint with minimum two years follow-up. Statistical analysis was performed with IBM SPSS software version 19 and the enumeration data were compared with Fisher’s exact test. P<0.05 was considered to indicate a statistically significant difference (95% CI). The DFS is defined as the time from treatment with trastuzumab until recurrence of tumor or death (for 3 years).

## Results

 In our study, the mean age of women with breast cancer at diagnosis was 46.39 ± 10.81 years (range, 24-78 years) (Table 1). Among 133 patients, 30 patients (22.6%) had metastasis and also 72 (54.1%) had right side involvement. Ninety three (69.9%) patients had lymph node invasion. Also, histological grade Ι, ΙΙ and ΙΙΙ were 9%, 66.2% and 24.8%, respectively. Size of tumor was divided into three groups that 26 patients (19.5%) had 0.1-2 cm, 85 (63.9) had 2.1-5 cm and the rest of patients had >5 cm. Ninety three patients (69.9%) had a lymph node invasion and for type of pathology, 127 patients (95.5%) had invasive ductal carcinoma (Table 1). Forty-five patients (33.8%) were classified as IHC 2+ and 88 (66.2%) were classified as IHC 3+. Fourteen IHC 2+ cases and seventy four IHC 3+ cases were found to be FISH positive (Table 2). We evaluated the concordance and discordance between IHC and FISH results for detection of Her2/neu protein.

**Table 1:** The Characteristics in women with breast cancer (N=133)

**Characteristics**	**N (%)**
**Age** Mean ± SD Range **Sex** Male Female **Metastasis** Yes No **Laterality** Right Breast Left Breast **Histological grade** Ι ΙΙ ΙΙΙ **Size of tumor in diameter** (cm) 0.1-2 2.1-5 >5 **Lymph node invasive** Yes No **Type of Pathology** Invasive ductal carcinoma Invasive lobular carcinoma	46.39 ± 10.81 24-78 0 (0) 133 (100) 30 (22.6) 103 (77.4) 72 (54.1) 61 (45.9) 12 (9) 88 (66.2) 33 (24.8) 26 (19.5) 85 (63.9) 22 (16.5) 93 (69.9) 40 (30.1) 127 (95.5) 6 (4.5)

The concordance rate is defined as the number of agreed to IHC 2+ and 3+ cases divided by the total number of IHC 2+ and 3+ cases. Also, the discordance rate is defined as the number of discrepant to IHC 2+ and 3+ cases (IHC 2+ or 3+ but Her-2 FISH negative) divided by the total number of IHC 2+ and 3+ cases. Concordance rates were 31.1% for IHC 2+ and 84.1% for IHC 3+. In other hand, discordance rates were 68.9% for IHC 2+ and 15.9% for IHC 3+ (Kappa=0.108, p<0.001) (Table 2).

Of 133 patients, 87 patients had age ≤50 years that 50 of them were ER positive, 43 were PR positive, 23 had IHC 2+ and 64 had IHC 3+. In the other hand, 46 patients had >50 years that 27 patients were ER positive, 30 were PR positive, 22 had IHC2+ and 24 had IHC3+(p>0.05) (Table 3).

**Figure 1:**
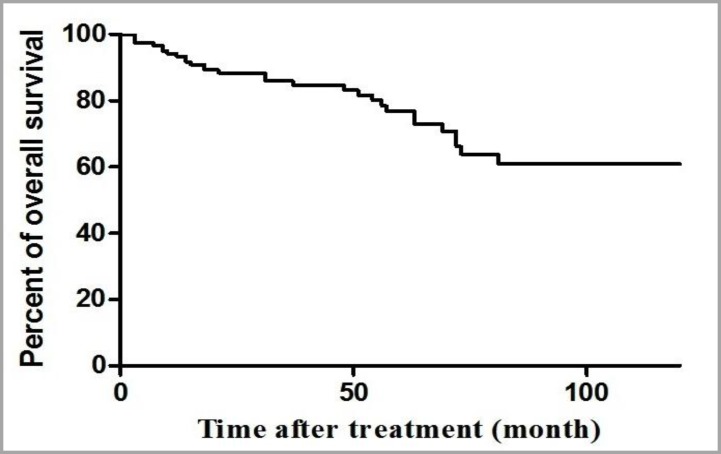
10 year overall survival for all of the patients (N=133)

**Figure 2:**
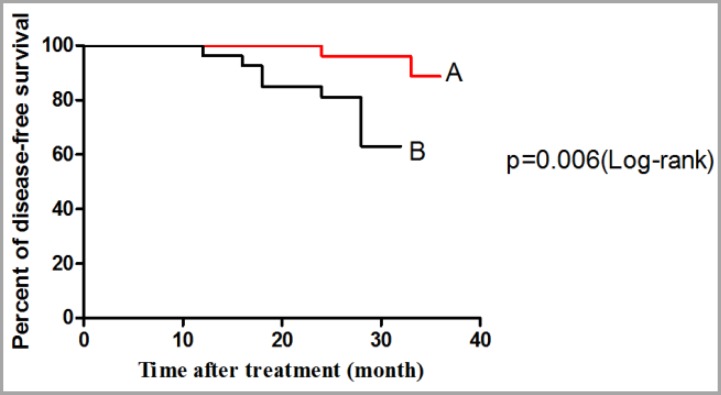
Disease-free survival from date of diagnosis Hre2 by immunohistochemistry: **(A)** for treated patients with trastuzumab, **(B) **treated patients without trastuzumab

Among 133 patients, 48 patients (36.1%) were treated with trastuzumab and 85 (63.9%) were treated without trastuzumab. Figure 1 shows the 10-year OS from date of diagnosis of disease in all of the patients. One way of summarizing survival data is to report the percentage of patients still alive at a fixed point in time. We might initially restrict our analysis to patients for whom we have complete information on the first two years of follow-up. In summary, after a two-year follow-up, 29 patients died and 92 patients were alive that 12 patients were lost follow-up before completing a two-year period and should therefore be excluded from the analysis. The 10-year survival rate was 70% and the mean survival was 49 months. Figure 2 shows the percentage of DFS, during 36 months from the date of treatment with trastizumab (the OS and the DFS based on IHC 2+, FISH+ and IHC 3+). Patients were excluded from the analysis that were lost follow-up before completing a two-year period of treatment (patients who were alive, but had not completed minimum 2 years of treatment period were excluded). Figure 2 (A) shows the DFS for patients treating with trastuzumab with the mean survival of 27.8 months and Figure 2B shows the DFS for patients treating without trastuzumab with the mean of 20.8 months (p=0.006, 95% CI of ratio= 0.031 to 0.561, hazard ratio=0.133). Patients died as a result of cancer or accident and natural death and patients in two groups that had treatment period less one year were excluded.

Furthermore, in the group of 29 patients who had IHC positive that were treated with trastuzumab for 3 years, one patient died, however in the group of 29 patients who never received trastuzumab, 8 patient died.

**Table 2:** Comparison of IHC and FISH results for detection of HER2

**IHC ** **scoring**	**Her-2 ** **FISH ** **amplified**	**Her-2 FISH ** **non-** **amplified**	**Concordance ** **by IHC**	**Discordance ** **by IHC**
2+ (n=45) 3+ (n=88)	14 74	31 14	14/45 (31.1%) 74/88 (84.1%)	31/45 (68.9%) 14/88 (15.9%)

**Table 3:** Correlation of markers expression with age

**Marker expression ** **(N)**	**Age≤50 years ** **(N=87)**	**Age>50 years ** **(N=46)**	**p-value**
HER2 2+ (45) HER2 3+ (88) ER (77) PR (83)	23 (51) 64 (73) 50 (65) 43 (52)	22 (49) 24 (37) 27 (25) 30 (48)	>0.05 >0.05 >0.05 >0.05

## Discussion

 Breast cancer is the most common malignancy among women.^10^ It is a leading cause of death in women. Adjuvant chemotherapy, commonly include alkylating agents and anthracyclines, improves survival rate in treated breast cancer.^11^ A study on 231 patients with breast cancer,^12^ showed that the mean age the patients was 45 years and also another study indicated that the mean age in the patients was 48.30 years.^13^

In our study the mean age was 46.39 years and was similar to results of other studies. HER2, a proto-oncogene, also known as c-erbB-2 or HER2/neu, located on chromosome 17q21, is considered to be closely associated with the occurrence and development of breast cancer and also IHC detection has become essential to many malignancies and plays a key role in tumor diagnosis, treatment and prognostic assessment.^14^ Therefore, the need for accurate detection of the Her2 alteration has now become even more important, because therapeutic decisions for patients with breast cancer are increasingly dependent on this information.^9^^,^^15^ This study evaluated the results of IHC and FISH among 133 patients with HER2 positive. There was an association between IHC 3+ and FISH. However, there was a discordance rate between IHC 2+ and FISH. This was similar to another study^9^ that reported FISH-positive and-negative rate for IHC2+ were 36 and 64%, respectively; 91 and 9% for IHC3+, respectively. This was statistically significant (p<0.01). A few studies,^16^^-^^18^ showed an association between IHC 3+ and no association between IHC 2+ with FISH.

Sui et al.^19^ and Park et al.^20^ showed an association between IHC 2+ and FISH. Therefore, an agreement between IHC and FISH is still controversial. The concordances in the cases of IHC 3+ are similar but there is a difference in the cases of IHC 2+. This difference may be due to the different sensitivity and specificity of the antibodies and probe used,^19^ or because of subjective interpretative variation in reporting, variable pre-analytical conditions, imperfect IHC methodology.^18^ ER and PR have important prognostic role in breast cancer and they also dictate the endocrine treatment, and important prognostic markers.^14^ Our study showed that in all of the patients, the positive rates of ER and PR were 54.8% and 61.6%, but results weren’t statistically significant (p>0.05). Her2+ reduces affection of treatment in the patients of ER and PR positive.

Several studies^14^^,^^21^^-^^23^ revealed the relationship of expression between subtypes (ER, PR and HER2) with age. ER and PR-high expressions were observed in female who were 50 or younger,^14^^,^^24^ such as this study. HER2-high expression was in female with >50 years,^14^ but we showed that it was in female ≤50 years such as result of Shomaf et al.^25^ There were no significant differences between the subtypes regarding age in present study. Trastuzumab was a humanized monoclonal antibody directed against the HER2/neu oncoprotein and has the ability to inhibit tumor growth in breast cancer patients overexpressing HER2.^26^ The mean survival of 48 treated patients with trastuzumab and 85 patients without it were 27.8 and 20.8 months. The five-year survival rate was 74.71% for patients with IHC 2+ and IHC 3+ that is favorable in comparison with previously reported series.^27^ The 10-year survival rate for our patients with IHC 2+ and IHC 3+, was 70% with mean survival 49 months. These results showed that the OS in our patients is better than other studies. The three-year DFS between two subgroups was statistically significant (p=0.006). The DFS rate for patients treating with trastuzumab and without trastuzumab was 93.3% and 76.6%, respectively. Therefore, trastuzumab therapy improves the survival of HER2-positive breast cancer patients.

## CONCLUSION

 We advise clinicians that FISH analysis is a predictive factor in breast cancer patients with IHC score 2+. In contrast, FISH analysis of score 3+ samples was not useful. Also, trastuzumab therapy is effective and tolerated for breast cancer with IHC 3+ and probably IHC 2+/FISH+.

## References

[1] Payandeh M, Shahriari-Ahmadi A, Sadeghi M (2016). Correlations between HER2 Expression and Other Prognostic Factors in Breast Cancer: Inverse Relations with the Ki-67 Index and P53 Status. Asian Pac J Cancer Prev.

[2] Hosseini MS, Arab M, Nemati Honar B (2013). Age - specific incidence rate change at breast Cancer and its different histopathologic subtypes in Iran and Western countries. Pak J Med Sci.

[3] Cui H, Cheng Y, Piao SZ (2014). Correlation between HER-2/neu(erbB-2) expression level and therapeutic effect of combination treatment with HERCEPTIN and chemotherapeutic agents in gastric cancer cell lines. Cancer Cell Int.

[4] Yarden Y, Sliwkowski MX (2001). Untangling the ErbB signalling network. Nat Rev Mol Cell Biol.

[5] Buendía JA, Vallejos C, Pichón-Rivière A (2013). An economic evaluation of trastuzumab as adjuvant treatment of early HER2-positive breast cancer patients in Colombia. Biomedica.

[6] Rexer BN, Chanthaphaychith S, Dahlman K (2014). Direct inhibition of PI3K in combination with dual HER2 inhibitors is required for optimal antitumor activity in HER2+ breast cancer cells. Breast Cancer Res.

[7] Banin Hirata BK, Maeda Oda JM, Losi Guembarovski R (2014). Molecular markers for breast cancer: prediction on tumor behavior. Dis Markers.

[8] Keyhani E, Muhammadnejad A, Behjati F (2013). Angiogenesis markers in breast cancer--potentially useful tools for priority setting of anti-angiogenic agents. Asian Pac J Cancer Prev.

[9] Bahreini F, Soltanian AR, Mehdipour P (2015). A meta-analysis on concordance between immunohistochemistry (IHC) and fluorescence in situ hybridization (FISH) to detect HER2 gene overexpression in breast cancer. Breast Cancer.

[10] Madani SH, Payandeh M, Sadeghi M (2016). The correlation between Ki-67 with other prognostic factors in breast cancer: A study in Iranian patients. Indian J Med Paediatr Oncol.

[11] Payandeh M, Khodarahmi R, Sadeghi M (2015). Appearance of Acute Myelogenous Leukemia (AML) in a Patient with Breast Cancer after Adjuvant Chemotherapy: Case Report and Review of the Literature. Iran J Cancer Prev.

[12] Erbil N, Dundar N, Inan C (2015). Breast cancer risk assessment using the gail model: a Turkish study. Asian Pac J Cancer Prev.

[13] Mohaghegh P, Yavari P, Akbari ME (2014). The Correlation between the Family Levels of Socioeconomic Status and Stage at Diagnosis of Breast Cancer. Iran J Cancer Prev.

[14] Qiao EQ, Ji M, Wu J (2013). Joint detection of multiple immunohistochemical indices and clinical significance in breast cancer. Mol Clin Oncol.

[15] Ha JH, Seong MK, Kim EK (2014). Serial Serum HER2 Measurements for the Detection of Breast Cancer Recurrence in HER2-Positive Patients. J Breast Cancer.

[16] Varga Z, Noske A, Ramach C (2013). Assessment of HER2 status in breast cancer: overall positivity rate and accuracy by fluorescence in situ hybridization and immunohistochemistry in a single institution over 12 years: a quality control study. BMC Cancer.

[17] Ghaffari SR, Sabokbar T, Dastan J (2011). Her2 amplification status in Iranian breast cancer patients: comparison of immunohistochemistry (IHC) and fluorescence in situ hybridisation (FISH). Asian Pac J Cancer Prev.

[18] Shirsat HS, Epari S, Shet T (2012). HER 2 status in invasive breast cancer: immunohistochemistry, fluorescence in-situ hybridization and chromogenic in-situ hybridization. Indian J Pathol Microbiol.

[19] Sui W, Ou M, Chen J (2009). Comparison of immunohistochemistry (IHC) and fluorescence in situ hybridization (FISH) assessment for Her-2 status in breast cancer. World J Surg Oncol.

[20] Park S, Park HS, Koo JS (2012). Breast cancers presenting luminal B subtype features show higher discordant human epidermal growth factor receptor 2 results between immunohistochemistry and fluorescence in situ hybridization. Cancer.

[21] DE Vargas Wolfgramm E, Gavioli CF, Entringer ML (2013). Histological profile and age at diagnosis of breast and ovarian tumors: A register-based study in Espirito Santo, Brazil. Mol Clin Oncol.

[22] Bertrand KA, Tamimi RM, Scott CG (2013). Mammographic density and risk of breast cancer by age and tumor characteristics. Breast Cancer Res.

[23] Sofi GN, Sofi JN, Nadeem R (2012). Estrogen receptor and progesterone receptor status in breast cancer in relation to age, histological grade, size of lesion and lymph node involvement. Asian Pac J Cancer Prev.

[24] Song Q, Huang R, Li J (2013). The diverse distribution of risk factors between breast cancer subtypes of ER, PR and HER2: a 10-year retrospective multi-center study in China. PLoS One.

[25] Shomaf M, Masad J, Najjar S (2013). Distribution of breast cancer subtypes among Jordanian women and correlation with histopathological grade: molecular sub classification study. JRSM Short Rep.

[26] Okita Y, Narita Y, Suzuki T (2013). Extended trastuzumab therapy improves the survival of HER2-positive breast cancer patients following surgery and radiotherapy for brain metastases. Mol Clin Oncol.

[27] Gultekin M, Eren G, Babacan T (2014). Metaplastic breast carcinoma: a heterogeneo disease. Asian Pac J Cancer Prev.

